# Estimating viral prevalence with data fusion for adaptive two‐phase pooled sampling

**DOI:** 10.1002/ece3.8107

**Published:** 2021-09-14

**Authors:** Andrew Hoegh, Alison J. Peel, Wyatt Madden, Manuel Ruiz Aravena, Aaron Morris, Alex Washburne, Raina K. Plowright

**Affiliations:** ^1^ Department of Mathematical Sciences Montana State University Bozeman MT USA; ^2^ Centre for Planetary Health and Food Security Griffith University Nathan QLD Australia; ^3^ Department of Microbiology and Immunology Montana State University Bozeman MT USA; ^4^ Department of Veterinary Medicine University of Cambridge Cambridge UK; ^5^ Selva Analytics LLC Bozeman MT USA

**Keywords:** adaptive sampling, Bayesian statistics, group testing

## Abstract

1. The COVID‐19 pandemic has highlighted the importance of efficient sampling strategies and statistical methods for monitoring infection prevalence, both in humans and in reservoir hosts. Pooled testing can be an efficient tool for learning pathogen prevalence in a population. Typically, pooled testing requires a second‐phase retesting procedure to identify infected individuals, but when the goal is solely to learn prevalence in a population, such as a reservoir host, there are more efficient methods for allocating the second‐phase samples.
2. To estimate pathogen prevalence in a population, this manuscript presents an approach for data fusion with two‐phased testing of pooled samples that allows more efficient estimation of prevalence with less samples than traditional methods. The first phase uses pooled samples to estimate the population prevalence and inform efficient strategies for the second phase. To combine information from both phases, we introduce a Bayesian data fusion procedure that combines pooled samples with individual samples for joint inferences about the population prevalence.
3. Data fusion procedures result in more efficient estimation of prevalence than traditional procedures that only use individual samples or a single phase of pooled sampling.
4. The manuscript presents guidance on implementing the first‐phase and second‐phase sampling plans using data fusion. Such methods can be used to assess the risk of pathogen spillover from reservoir hosts to humans, or to track pathogens such as SARS‐CoV‐2 in populations.

## Graphical abstract

Pooled testing is a common procedure for efficient sampling to determine population prevalence and identify positive individuals. When the goal is to learn population prevalence, without regard for identifying positive individuals, data integration results in more precise estimates than retesting individuals in positive pools.

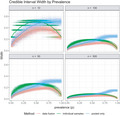

## 1 INTRODUCTION

The rapid pandemic spread of COVID‐19 has overwhelmed health systems globally, from funding and supply chains to testing and hospital capacity. The capacity to detect infections circulating in a population is constrained by technical limitations, costs, and logistics, which rapidly scale with the temporal and spatial scales of epidemics. The COVID‐19 pandemic has highlighted the importance of efficient sampling strategies and statistical methods for monitoring infection prevalence, both in humans and in reservoir hosts. In studies of reservoir hosts, the research question is not necessarily whether an individual is infected, but rather the goal can be to estimate the prevalence in the reservoir host population, and how this changes over space and time. Furthermore, funding for screening potential reservoirs is generally limited compared with human screening. Nevertheless, estimating prevalence in reservoir hosts is critical for understanding drivers of pathogen spillover and precise estimates of population prevalence require testing a large number of samples (Plowright et al., 2017, 2019). Unfortunately, the total number of samples that can be screened is limited by the high costs of field sampling, laboratory testing, and other fiscal constraints; thus, strategies to optimize testing samples are critical for successful disease surveillance.

One approach for testing, particularly when the prevalence rates are expected to be low, is to pool individual samples to assess whether one, or more, of the pooled samples results in a positive test. This pooling procedure is commonly referred to as group testing (Du et al., 2000). Group testing was first developed in 1940s to detect cases of syphilis in soldiers in the US military during the Second World War. The technique increases efficiency of utilization of limited resources during outbreaks or surveillance programs with direct impact on response capacity (Dorfman, 1943). However, the effectiveness of pool testing can be compromised as disease incidence and prevalence increases, as this results in more tests conducted during the second rounds of diagnostic assays to identify individual positive samples. Thus, there is a need for clear guidance on the optimal number of samples to pool or number of total pools to be tested. Depending on the population prevalence, pooling too many or too few samples can decrease precision in the estimated parameter and make inferences on the population prevalence unreliable, or require multiple stages of individual testing.

In light of testing limitations, pooled techniques have been adapted for COVID‐19 screening in humans (Mallapaty, 2020; Mutesa et al., 2020). In this scenario, the primary intent is to determine which individuals are infected to implement isolation of cases and contact tracing protocols to mitigate spread of the virus. The idea is to first combine individual samples into a single pooled sample. If the pooled sample is negative, then all of the individual samples are assumed to be negative. If a pooled sample tests positive, although it is not immediately clear how many and which of the individual tests are positive, there are many strategies to subsequently identify this.

The simplest strategy involves retesting each of the individual samples that comprise the pool. This procedure will enable a researcher to have a complete dataset that identifies all individuals that test positive. Rather than automatically retesting all individuals in a positive pool, Sobel and Elashoff (1975) present a hierarchical approach for testing subsets of the pools in an iterative fashion. Phatarfod and Sudbury (1994) proposed an array approach where an individual specimen was divided across multiple pools. In the context of estimating population prevalence, Bilder et al. (2010) present “Informative Retesting,” where the retesting approach uses individual covariate information in the retesting protocol and Hepworth and Watson (2017); Hepworth and Walter (2020) present a restricted randomization approach for retesting similar in spirit to Phatarfod and Sudbury (1994)’s arrays. With appropriate pooling strategies and retesting positive pools, the overall number of samples tested can be less than the total number of individuals.

In addition to testing humans, there are also broad efforts to identify the reservoir hosts for SARS‐CoV‐2‐related viruses, and coronaviruses more broadly. In contrast studies where the goal is case identification (Zhang et al., 2013), however, individual results may not be required and population‐level estimates of prevalence are often sufficient to identify hosts and understand transmission dynamics within their populations. In some cases, this will involve collecting new samples with purpose‐fit sampling designs, or in other cases, sample banks may already exist that can be screened for coronaviruses. The number of samples available does not always match the funding available for screening, and optimal testing approaches to achieve desired inferences are required. Thus, to understand population prevalence in reservoir populations and in other situations where the focus is on population prevalence, we propose a data fusion procedure, with pooled testing, that enables estimates of population prevalence in multiphase studies without retesting positive pools.

With the remainder of this article, Section 2 details sampling approaches for screening individuals and presents estimation approaches, including data fusion techniques, for estimating population prevalence, Section 3 contains results from a set of simulation studies, and Section 4 concludes with a discussion.

## 2 MATERIALS AND METHODS

### 2.1 Sampling approaches

In some scientific studies, particularly for studying viral prevalence in reservoir hosts, a large number of samples may already exist or can be collected for minimal costs relative to the cost of testing samples. Ideally, all of these samples would be tested; however, with this work, we assume that the number of samples that can be tested is constrained. Most often the constraint is the research funding, but limits can also be a result of instrument capacity or availability of materials. Given these constraints, a sampling strategy needs to be devised to determine which individual samples to test and whether these samples should be combined into pools.

The optimal number of samples per pool requires knowledge of the population prevalence—which is generally unknown for investigations into novel host–pathogen combinations. Thus, an approach for pooled sampling is to implement a two‐phase sampling design where an initial pooling in the first phase can be used to inform second‐phase sampling strategies. Altering the pool size or testing additional samples individually in the second phase requires a data fusion procedure to combine inferences across pooled and individual samples. With this work, we provide guidance on adaptive two‐phase sampling designs while combining pooled samples with individual samples using a novel Bayesian data fusion (Allen, 2017) procedure. We will show that this procedure results in a more precise estimator of the population prevalence than retesting positive pools.

#### 2.1.1 Pooling and group testing

If the overall population prevalence is close to zero, then most of the individual samples will be negative, and therefore, testing costs per positive sample detected are high. With a fixed cost for a single test, pooling strategies allow two or more samples to be jointly tested for the same cost as an individual sample. While a negative pool implies that all of the individual samples are negative, a positive pooled sample only implies that one or more samples in the pool are positive. The positive samples can be retested or be directly used to inform population prevalence and future sampling. Optimal pooling approaches require knowledge of population prevalence. This article focuses on data fusion methods for combining pooled results from multiple phases without requiring retesting positive pools to estimate population prevalence and establish optimal pool sizes, which can include either retesting samples from positive pools; testing additional pools of the same or different sizes; or testing additional individual samples, which requires a data fusion procedure.

Pooling procedures involve combining two or more individual samples into a pool. There is a long history of pooled sampling approaches (Bhattacharyya et al., 1979; Burrows, 1987; Dorfman, 1943; Swallow, 1985) and associated statistical methodology for parameter estimation (Biggerstaff, 2008; Chen et al., 2009; Colón et al., 2001; Hepworth, 2005). The difference between these approaches and what we propose is that existing methods are generally focused on designing a single‐phase pooled sampling plan or estimating population prevalence conditional on pooled samples (Colón et al., 2001). In contrast, we propose a procedure that combines adaptive pooling with data fusion techniques to integrate pooled and individual samples combining.

#### 2.1.2 **Adaptive sampling** **+ two‐phase sampling**

Adaptive sampling is a procedure where the sampling strategy is informed by previously collected data. Adaptive sampling has a history in quality control fields (Prabhu et al., 1994; Runger & Montgomery, 1993; Runger & Pignatiello Jr, 1991). More recently, adaptive sampling has become popular in sensor networks (Gedik et al., 2007; Jain & Chang, 2004) and Bayesian model selection procedures (Clyde et al., 2011; Nott & Kohn, 2005). In the context of pooled testing, Hepworth (1996) proposed a sequential approach for pooled sizes. In theory, the number of samples in a pool could be adaptively changed after each test (e.g., as an outbreak is developing and cases rise and then subsequently falls); however, for practical implementation we restrict the sampling procedure to two phases. In addition to the sampling approach, there is a corresponding estimation problem, often referred to as sequential estimation (Lai, 2001). Estimation methods for the two‐phase sampling approach are described in the next section.

In ecological settings, due to data collection, processing, and analysis procedures, adaptive sampling and estimation are generally conducted with a small number of discrete phases. Often, two‐phase approaches include an initial screening of rare species that informs more efficient resource allocation in the second phase (Pacifici et al., 2012). In other settings, certain types of sampling are more expensive and a cheaper method is used for an initial screening before employing a more expensive sampling procedure (Rivest et al., 1990; Villella & Smith, 2005; Villella & Smith, 2005). Conceptually, pooled sampling represents a cheaper form of sampling, on a per sample basis, and hence is quite similar to these scenarios.

Adaptive sampling approaches have been developed for monitoring prevalence, both with and without pooling. Reilly (1996) developed optimal sampling approaches for two‐phase sampling where the cost of sampling may differ between phase 1 and phase 2. Breslow and Chatterjee (1999) presented an approach for two‐phase sampling where the second‐phase estimation is a case–control sample. McIsaac and Cook (2015) developed a framework of two‐phase sampling which they define as “response‐dependent,” meaning that sampling approach in the second phase depends on the responses in the first phase. However, we are not aware of any approaches that combine adaptive sampling with data fusion for estimation of viral prevalence by enabling researchers to combine information from pooled and individual samples. Our two‐phase approach would be beneficial in calibrating the optimal pool size in any biological setting with pooled samples, such as pooled samples for Salmonella detection (Kinde et al., 1996) or estimating prevalence of infected insect vectors (Ebert et al., 2010). Moreover, the ability to fuse pooled and individual samples can lead to more efficient sampling in any scenario that implemented a phase one pilot study to inform sampling parameters for prevalence or took an either or approach to pooled sampling versus individual samples (Arnold et al., 2011).

#### 2.1.3 Choosing pool size and identifiability of population prevalence

When choosing pool sizes, one issue is identifiability of population prevalence when the rate of prevalence is high and a large number of samples are pooled. The maximum‐likelihood estimator of the prevalence has been shown to be biased (Colón et al., 2001). The reason is that the probability that a pooled sample tests positive, is π,

(1) $$\pi =\left(1‐{\left(1‐p\right)}^{n}\right)$$

can be effectively 1 for a range of population prevalence, which is denoted with *p*. For illustrative purposes, assume that *p* > .5 and the pool size, *n*, is 10. In this example, if *p* = .5, then *π* = 0.999. Thus, nearly all pooled samples would test positive for prevalence between 0.5 and 1, and hence, it would be impossible to differentiate values in the interval between 0.5 and 1, or in other words, this is not identifiable. This issue is further magnified when all, or nearly all, of the pooled samples test positive. Hepworth and Watson (2009) present a bias correction method for maximum‐likelihood approaches of estimating prevalence. Note that Bayesian estimates, particularly with informative prior distributions, are better suited for this problem. In fact, Hepworth and Watson (2009) mention one possible solution for calculating the MLE with all positive samples is “to calculate the expectation of the posterior distribution of *p* under an appropriate prior distribution.” Of course, retesting all positive pooled samples would also enable a relative frequency calculation of prevalence.

Implicitly the calculation of *π* in Equation 1 assumes that the samples are independent. For the applications we are primarily focused on, viral surveillance in wildlife populations where individual samples can be randomly assigned to pools, this is usually a reasonable assumption. However, if this assumption is violated, Equation 1 would not be valid. If the goal is to follow up with individual samples, such as the COVID‐19 scenario in humans, correlation could be exploited for more efficient groupings. For example, a group consisting of members of a household or wildlife populations with spatial structure would be more likely to be all positive or negative, than a random selection of individuals. Having pools that are all negative or mostly positive, due to correlation from patients interacting and potentially infecting each other, would reduce the total number of tests.

### 2.2 Model framework

Using two‐phase adaptive sampling, we describe three methods for estimating population prevalence using a combination of pooled and individual samples. Using information from data in the first phase to inform the second phase, efficient adaptive sampling requires sequential estimation. These estimation methods present a set of approaches for two‐phase sampling. Bayesian data fusion methods are implemented to estimate overall prevalence, where a beta(*α*,*β*) distribution is used as the prior for *p*. For the experiments in Section 3, a uniform prior, beta(1,1) is used, but for specific applications, subject matter expertise can, and should, be used to select parameters in the beta distribution for an informed prior of population prevalence. Practitioners with knowledge of prevalence can use established prior elicitation techniques, such as Wu et al. (2008), to create informative prior distributions.

Data integration is formally defined by using different streams that measure the response of interest to make combined inferences and uncertainty calculations. It is important to differentiate between collecting streams of data that can be used as covariates with that of multiple streams of data about the outcome of interest. In the former, information like demographic information could be collected that might be indicative of the parameter of interest. In contrast, the latter scenario contains multiple direct measurements of the outcome of interest, but they may be on different spatial and/or temporal scales and require a formal method for combining the data. In this work, we use a more general term data fusion, rather than data integration, to signify the combining pools of different sizes, including individual samples. Data from these settings consist of positive or negative results from pools of different sizes, potentially along with individual results. Following the framework defined in Miller et al. (2019), we evaluate a *joint likelihood* method for combining the data streams.

#### 2.2.1 Individual samples only

If all samples are tested individually, estimating the population prevalence is straightforward. The individual samples are modeled with a Bernoulli distribution,

(2) $$Y_{i}∼\text{Bernoulli}\left(p\right),$$

where *Y_i_* is a binary variable denoting whether the *i*th sample tests positive and *p* is population prevalence or equivalently the probability that an individual tests positive. Given the sampling model in Equation 2 and the beta prior distribution for *p*, the posterior distribution for *p* has a closed form. Specifically, the posterior distribution is

(3) $$p|Y∼\text{beta}\left(\alpha +\sum_{i}y_{i},\beta +n‐\sum_{i}y_{i}\right),$$

where ***Y*** = (*Y*_1_,…, *Y_n_*) and *n* is the total number of individuals that are tested.

#### 2.2.2 Pooled samples

With pooled samples, a common second‐phase approach is to retest individuals from pools that test positive. Brookmeyer (1999) presents a conditional probability function that accounts for the dependence between the initial and subsequent pools. When the retesting pools are of size one, the end result is that the total number of positives and negatives are known and the exact posterior distribution can be computed using Equation 3. If the prevalence is low, this approach can be more efficient than testing individual samples. In particular, this approach is more efficient, on a per‐test basis, if the expected number of samples tested is less than the total number of individual samples.

Rather than a set of individual tests, the sampling model, or the statistical likelihood, is defined as:

(4) $$Z_{j}∼\text{Bernoulli}\left(\pi_{j}\right)$$

(5) $$\pi_{j}=\left(1‐{\left(1‐p\right)}^{n_{j}}\right)$$

where *Z_j_* is a binary variable denoting whether the *j*th pooled sample tests positive, *n_j_* is the number of samples in pool *j*, and *π_j_* is a function of *p* that corresponds to the probability that a pooled sample *j* tests positive. The posterior distribution for *p* does not have a closed‐form solution as the likelihood is a nonlinear function of *p*. Rather, this requires Markov chain Monte Carlo (MCMC) techniques. A Metropolis–Hastings algorithm, which is detailed in Appendix 1, is used to estimate the prevalence, *p*, using the calculated probability π*_j_*.

#### 2.2.3 Integrated analysis

When the data consist of pooled and individual samples, a data fusion approach combines two different streams: the individual samples, ***Y***, and the pooled samples, ***Z***, where ***Z*** = (*Z*_1_,…,*Z_J_*) is the collection of *J* pooled samples. It is important to note that the individual samples are not contained in the pools, but rather could come from a different data collection process. Then, using a joint likelihood method as in Miller et al. (2019) they can be modeled as.

(6) $$Y_{i}∼\text{Bernoulli}\left(p\right)$$

(7) $$Z_{j}∼\text{Bernoulli}\left(\pi_{j}\right)$$

(8) $$\pi_{j}=\left(1‐{\left(1‐p\right)}^{n_{j}}\right).$$

Similar to pooled samples scenario, a Metropolis–Hastings algorithm is used to estimate the prevalence, *p*. The difference is that the posterior distribution of *p* is now conditional on both the pooled samples (***Z***) and the individual samples (***Y***).

(9) $$p\left|Z,Y\propto Y\right|p\times Z|p\times p.$$

Formally, the joint likelihood approach uses the likelihood of the individual samples, ***Y*** |*p*, and the pooled samples, ***Z*** |*p*. The joint likelihood is proportional to Equation 9. The MCMC algorithm for this data fusion is detailed in Appendix 1.

## 3 RESULTS

A set of four synthetic studies are constructed to explore adaptive sampling and data fusion techniques across Phase 1 (initial testing of samples to obtain initial estimates of prevalence) and Phase 2 (follow‐up or additional testing to obtain more detailed prevalence estimates). Simulation 1 provides justification for the data fusion procedure against pooled samples or individual samples alone for Phase 1 testing. Simulation 2 shows the data fusion procedure with a phase 2 plan that tests new individual results in a more efficient estimation following up individuals in pools that tested positive. Simulation 3 contains a demonstration to inform the phase 1 pooling procedure. Simulation 4 evaluates a set of phase two approaches, given a phase pooling. All simulations use 99 prevalence values, 0.01 to 0.99, with 200 replications for each prevalence value. MCMC are run for 10,000 iterations with the first 1,000 iterations discarded as burn‐in. Prevalence values are initialized at 0.5 for each of the simulations. Code for recreating the simulations or adjusting pool size/sample size is available in the supplemental materials section.

### 3.1 Simulation 1: Efficacy of data fusion for phase 1 of testing

The purpose of the first simulation study is to show the efficacy of integrated analysis against individual or pooled samples alone. The study compares three modeling approaches for a fixed number of samples. Specifically, there are 100 pools of samples, with each pool comprising three individual samples, along with another 100 individual samples that are tested. The first modeling approach uses just the individual data, the second uses just the pooled samples, and the third uses both pieces of information in a data fusion framework.

**FIGURE 1:**
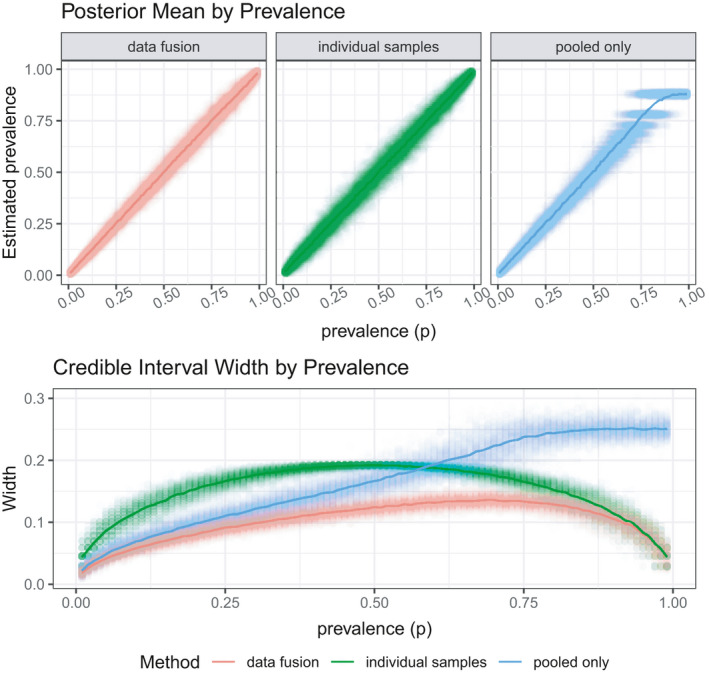
Simulation 1: Data fusion efficacy for phase 1 testing. This simulation shows that data fusion technique, which requires combining pooled and individual samples, is superior to either approach alone. For the lower ranges of prevalence, the posterior mean of all of the methods tracks the overall prevalence, on average. However, as the prevalence becomes closer to one, the pooled only approach is unable to identify the true prevalence. The data fusion approach has the narrowest credible interval width across a range of prevalence values

The top panel of Figure 1 shows the posterior mean for all of the methods across a range of prevalence values. With only three samples per pool, the pooled approach tracks the true prevalence values fairly well for lower prevalence values, but as the prevalence approaches one, estimates of the prevalence are biased. This is due to the difficulty of identifying higher levels of prevalence from pooled samples, as discussed above. This bias would be amplified with larger pool sizes. Both the data fusion and individual samples do a good job of identifying the true prevalence; however, the integrated approach provides, on average, more accurate estimates of the true prevalence.

The bottom panel of Figure 1 shows the average width of a 95 percent highest posterior density credible interval for each method across a range of prevalence values. For lower levels of prevalence, 100 pooled samples, of size 3, result in more efficient estimation of overall prevalence than 100 individuals. However, as the prevalence increases, the individual samples become more efficient. The data fusion approach, which combines both the individual samples and the pooled samples, has a smaller credible interval width for the entire prevalence range than both other approaches. This is unsurprising as more data are used, but this verifies the utility of the data fusion method that we propose. The data fusion approach has smaller credible interval width than both other methods and also provides accurate posterior mean estimates.

**FIGURE 2:**
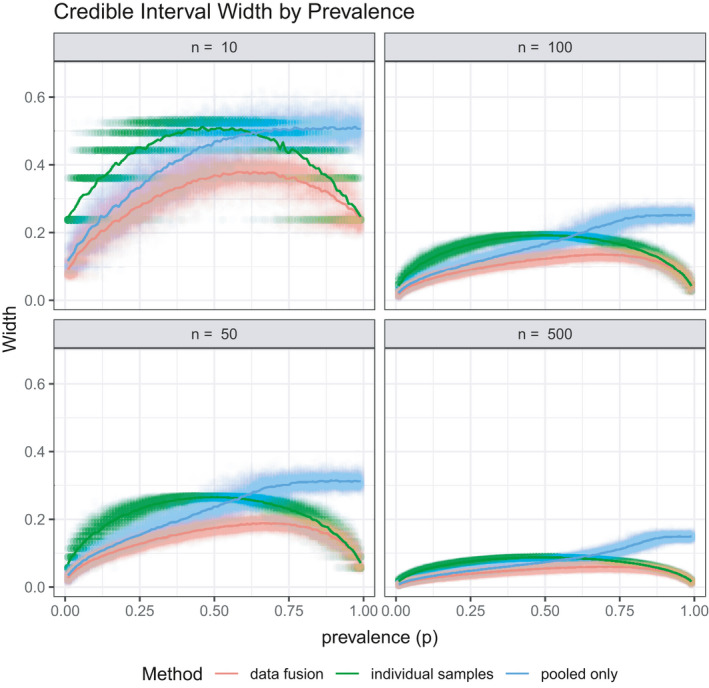
Simulation 1: Data fusion efficacy for phase 1 testing. Credible interval width shown for a set of sample sizes that correspond to the total number of pools and individual samples

The results from Figure 1 corresponded to 100 samples, both for the pooled samples (of size 3) and individual samples. Figure 2 shows the credible interval width for a set of sample sizes. Unsurprisingly, the credible intervals are narrower for larger sample sizes, but this figure will illustrate the number of total samples required for achieving target credible interval width. Additionally, code for recreating the simulation, and adjusting sample size and/or pool size, is available in the supplemental materials.

### 3.2 Simulation 2: Retesting versus new samples for phase 2 of testing

Following the initial phase of testing to obtain an estimate of population prevalence, this next simulation explores a pair of methods for phase two of testing: retesting all samples in a positive pool or testing a new set of individual samples and using our data fusion framework. To make the comparison “fair,” the retesting procedure is done first and then the same number of samples is used in the data fusion approach. For instance, if the retesting procedure results in a total of 35 phase two tests, then 35 additional samples are tested (without pooling) for the data fusion procedure. The data fusion procedure then combines inferences from the original pooled samples and the new individual samples.

**FIGURE 3:**
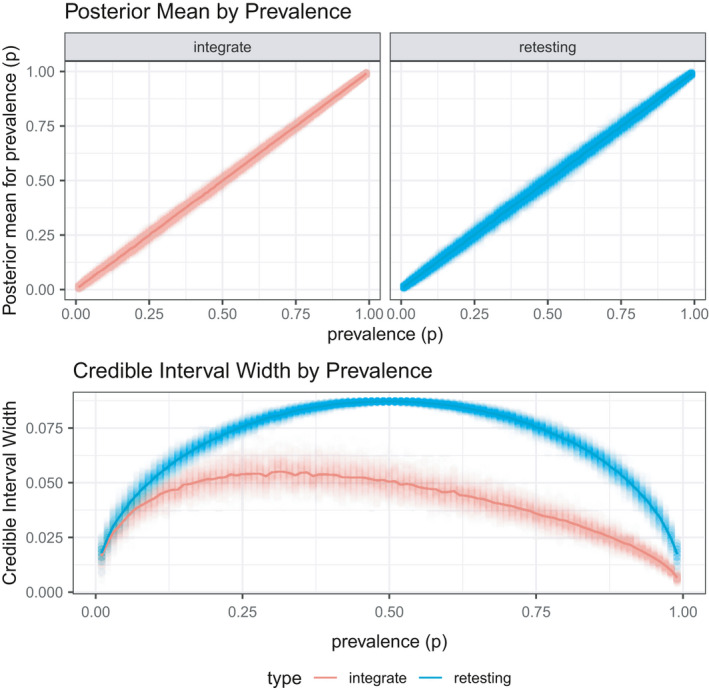
Simulation 2: Retesting versus new samples for phase 2 testing. While the posterior mean for both approaches is accurate, the data fusion procedure has a substantially smaller credible interval width for the entire range of prevalence values for the same number of tests conducted

**FIGURE 4:**
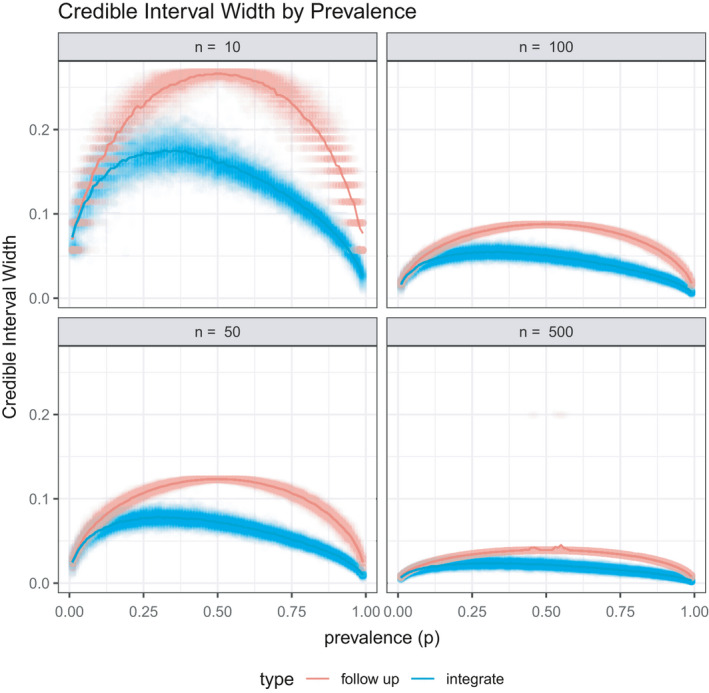
Simulation 2: Retesting versus new samples for phase 2 testing. Credible interval width shown for a set of sample sizes that correspond to the total number of tests conducted

The top panel of Figure 3 shows that both retesting positive pools and a data fusion approach result in accurate posterior means. However, in the bottom panel of Figure 3, the data fusion procedure has smaller credible interval width for all prevalence levels. This result is fairly intuitive as taking additional samples would contain more information that retesting old samples, where it is known that at least one sample is positive Figure 4 shows the credible interval width as a function of sample size. Prior to this work, the challenge had been the lack of a coherent method to combine the pooled and individual samples, which our proposed data fusion procedure allows.

### 3.3 Simulation 3: Initial pool sizes for phase 1 of testing

Simulation 1 assumed a pool size of three samples per pool for phase one of testing; however, given the importance of choosing the pool size in phase one, the third simulation compares different pooling sizes across a range of prevalence values from 0.01 to 0.99. While pools of size three are useful for higher prevalence values, near 0.5, with lower prevalence values larger pools should be considered. The phase one goal is to explore the posterior means and the widths of the credible intervals for different pooling sizes to inform phase 2 decisions. This simulation assesses pool sizes of 1, 3, 5, 7, and 9 to compare the posterior means and credible interval width as a function of the prevalence rate.

**FIGURE 5:**
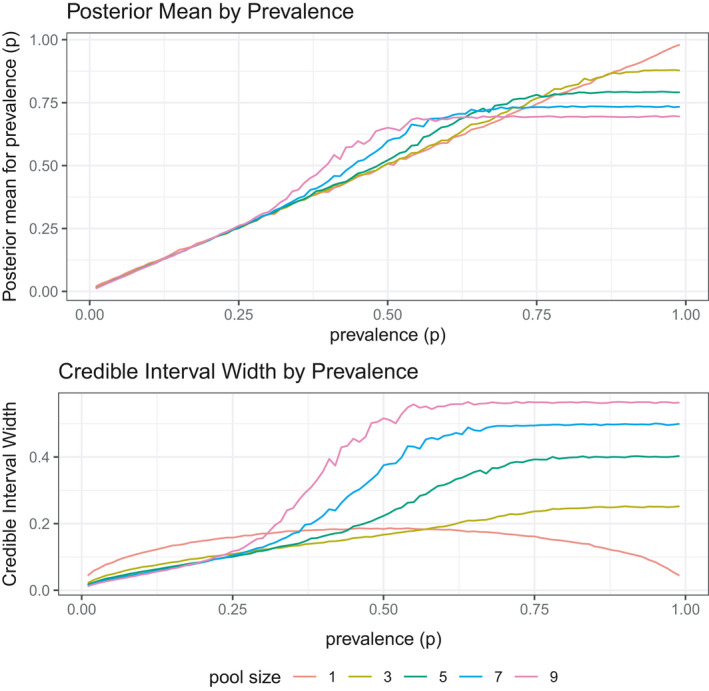
Simulation 3: Initial pool sizes for phase 1 of testing. With prevalence values close to zero larger sized pools have smaller credible intervals; however, as the prevalence gets larger, the pooled samples become inaccurate and imprecise. Initial pool size can be selected based on mean and credible interval width for a priori prevalence belief

Similar to simulation 1, Figure 5 contains the posterior means and credible intervals for each of the comparison methods. When the prevalence is close to zero, all of the pools accurately estimate the true prevalence. The larger pool sizes have a more precise estimate, which is not surprising as the posterior variance, or similarly the standard error of the point estimate, is a function of the total number of individual pooled samples. However, as the prevalence gets larger, the posterior means of the pooled estimators start to deviate from the true prevalence. In fact, at a certain threshold when all of the pooled samples end up positive, the posterior means flatten off and the posterior distribution ends up being uniform between a threshold of *p* values that map to *θ* ≈ 1. In addition to inaccurate posterior means, this identifiability problem also results in credible intervals that are very wide.

The takeaway from this simulation is that a larger pool size can be more efficient with lower prevalence. The potential drawback to large pool sizes is the case when prevalence is high and many, or all, of the pools test positive, resulting in an imprecise answer. Prior knowledge about the prevalence should be used in selecting phase 1 pooling strategies. As described in Hepworth and Watson (2009), researchers should give thought to the upper level of population prevalence, by estimating the probability of prevalence exceeding a specified threshold, when choosing pool sizes. The calculations in Section 2.1.3 provide guidance on the largest prevalence that can be identified from pooled samples. In spite of this issue, simulation 4 builds on this scenario by exploring a set of methods for phase 2 estimation that can correct some of the problems with suboptimal phase 1 pool sizes.

### 3.4 Simulation 4: Adaptive sampling for phase 2 of testing

Simulation 3 provided guidance on choosing the phase 1 pool size; this simulation follows that to compare the performance of phase 2 sampling and estimation techniques. Simulation 2 already established that the second‐phase data fusion procedure (adding data from new individual samples without pooling) is superior to following up with retesting of individual samples in positive pools. For this next scenario, we compare outcomes when the second‐phase data fusion also uses pooled samples. Specifically, in simulation 4, phase 1 consists of 100 pooled samples of size 5; then, we compare second‐phase testing with pools of sizes 1, 3, and 5 that run the same number of total tests.

**FIGURE 6:**
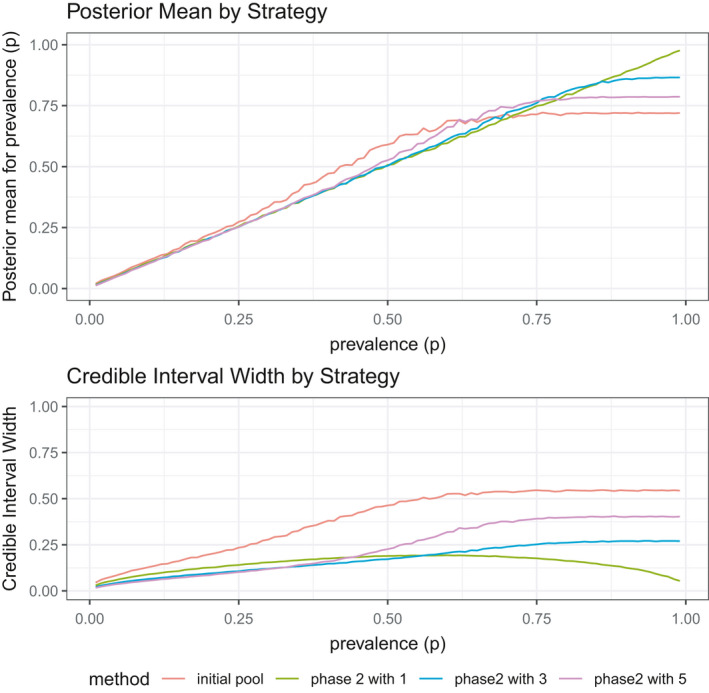
Simulation 4: Adaptive sampling for phase 2 of testing. Phase 1 credible interval constructed from 20 pooled samples of size 5. Depending on the prevalence, which can be partially inferred from the phase 1 samples, phase 2 plans should differ

Figure 6 shows the estimated posterior means and credible interval widths from the phase 1 samples as well as the three phase 2 approaches. The only approach that accurately estimates the entire range of prevalence values is the phase 2 method that tests additional single samples, as taking additional pools of size 3 or size 5 results in inaccurate estimates with larger prevalence values. Taking individual samples also has the smallest credible interval with large prevalence values. Otherwise, with smaller prevalence values, varying levels of pooled samples can result in accurate estimates and precise results.

Given phase 1 results, p can be estimated using Equations 4 and 5 along with the associated MCMC procedure. Then, this result can be used to identify appropriate phase 2 approaches. If the prevalence value from the phase 1 approach is estimated to be close to 1, individual samples should be used. With prevalence values closer to zero, additional pooled samples can give accurate and more precise results. As the prevalence values get closer and closer to zero, larger pool sizes can give the most precise results with accurate estimation.

### 3.5 Conclusion

The simulation studies have verified the utility of combining data fusion with two‐phase adaptive sampling. By introducing data fusion methods that combine individual and pooled samples, estimates of prevalence are more efficient, and more accurate, than using just the individual or pooled samples alone. Pooled samples are commonly used to screen sets of individuals, where the individuals that compose pools that test positive are retested. If the goal is to estimate population prevalence, rather than identify individuals that are positive, testing new individuals, and using data fusion methods, is a more efficient procedure than retesting individuals in positive pools.

Optimal decisions for pooled sampling strategies to estimate population prevalence require knowing the population prevalence. While that is not possible, data fusion methods enable estimating population prevalence from the first‐phase samples to inform second‐phase adaptive sampling techniques. The data fusion methods that we have presented are the necessary component to combine information across pooled results of different sizes, including potentially individual samples. Prior information can be useful in designing pool sizes for phase one sampling. When considering phase two sampling strategies, population prevalence can be estimated, to a degree, from the phase 1 samples. When the prevalence is larger, smaller pool sizes, or even individual samples, should be used. When prevalence is smaller, larger pool sizes can be used to increase efficiency without compromising accuracy.

## 4 DISCUSSION

When the goal is to estimate population prevalence, rather than identify infected individuals, pooled testing without retesting positive pools as a second‐phase strategy can result in fewer overall tests or more efficient estimates than testing individual samples. Sampling strategies for pooled testing require selecting the pool size as well as the total number of pools to test. Selecting optimal, or efficient, pool sizes is a well‐known problem (Thompson, 1962) that requires knowing the population prevalence.

As the population prevalence is what researchers are trying to understand, it is typically unknown and cannot be directly used for pool size selection. Thus, there is a trade‐off when considering the choice between larger or smaller pools. Pool sizes that are too small result in less efficient estimation. Pool sizes that are too large result in inflated credible intervals and inaccurate estimation. However, another approach is to implement a two‐phase sampling procedure that can be used to gain information about the population prevalence, which can then be used to inform pool sizes for the second phase and subsequent testing.

The two‐phase approach can enable researchers to learn about the population prevalence before conducting all of the tests on a given pool size; however, using data from the first phase and second phase to jointly inform population prevalence requires formal data fusion procedures. This article presents data fusion methods that can combine information from pooled samples of different sizes, including individual samples.

Adaptive sampling could be implemented on a sample‐to‐sample basis where the pool size changes with each test. However, in many settings this is impractical as tests are run in large batches or concurrently. In general, a smaller share of the total tests should be allocated to the phase 1 setting than the phase 2 setting. This allows the information gained from phase 1 to be used to create the most efficient pool sizes.

This work has assumed perfect sensitivity and specificity of tests. In practice, this is rarely the case. However, known sensitivity and specificity could easily be incorporated into the model framework to adjust for the potential of false positive and false negatives. If the sensitivity and specificity of the pooled tests depend on the proportion of pooled samples that are positive or negative, this could also be handled in the model framework but would likely require laboratory testing to understand the relationship between sensitivity and specificity and the underlying samples.

One potential advantage of the data integrated approach and an active area of ongoing research is the ability to incorporate individual‐level covariate information, see McMahan et al. (2017) or Joyner et al. (2020). Consider the case that demographic information is available and is related to the prevalence of an individual. Then, pooled tests could be used to inform the overall prevalence in the population, along with the individual samples. The individual samples also can be used to infer the relationship between individual demographic information and the likelihood of being infected.

## CONFLICT OF INTEREST

We declare no conflicts of interest.

## AUTHOR CONTRIBUTIONS

**Andrew Hoegh:** Conceptualization (equal); methodology (lead); software (lead); writing—original draft (lead); writing—review and editing (equal). **Alison J. Peel:** Conceptualization (equal); writing—review and editing (equal). **Wyatt Madden:** Methodology (supporting); software (supporting); writing—review and editing (supporting). **Manuel Ruiz Aravena:** Conceptualization (supporting); writing—review and editing (equal). **Aaron Morris:** Writing—review and editing (supporting). **Alex Washburne:** Writing—review and editing (supporting). **Raina K. Plowright:** Conceptualization (equal); funding acquisition (lead); writing—review and editing (equal).

## Supporting information

Supplementary Material

Supplement

Supplementary Material

## Data Availability

No new data are created for this project; however, code for the simulations highlighted in the manuscript is publicly available on github (https://github.com/andyhoegh/DataIntegration).

## APPENDIX 1

### MCMC for pooled and integrated analysis

The MCMC details for both the pooled and integrated analysis are quite similar, with the only difference being the target function, *f*(). Furthermore, the MCMC approach is basically a standard Metropolis–Hastings algorithm with an additional transform step to convert *p* to *π*.

where *g*() is a random walk proposal function. For pooled samples, *f*() is Equation 4 combined with the prior distribution for *π*. For the data fusion approach, *f*() is Equation 9.
