# Supplementary material for: Estimating viral prevalence with data fusion for adaptive two‐phase pooled sampling
Source: Ecol Evol. 2021 Sep 14;11(20):14012–23. doi: 10.1002/ece3.8107 (PMC8525136; doi:10.1002/ece3.8107)
Supplement: Supplementary file 1 — Supplementary Material [file ECE3-11-14012-s002.pdf]

Estimating Viral Prevalence with Data Fusion for Adaptive Two-Phase Pooled Sampling

Initialization

```
# Quicker Simulations
num_replicates <- 20
theta_seq <- seq(0.01, .99, by = .05)

# Settings from Paper, may take a while to compile
#num_replicates <- 200
#theta_seq <- seq(0.01, .99, by = .01)
```

Simulation 1

Part A: N = 100

```
set.seed(07012020)
num_pooled_samples <- 100
num_indiv_samples <- 100
pool_size <- 3
num_theta <- length(theta_seq)

y_replicates <- array(0, dim = c(num_theta,num_replicates, 2))
z_replicates <- array(0, dim = c(num_theta,num_replicates, 2))
yz_replicates <- array(0, dim = c(num_theta,num_replicates, 2))
y_mean <- y_width <- matrix(0, num_theta, num_replicates)
z_mean <- z_width <- matrix(0, num_theta, num_replicates)
yz_mean <- yz_width <- matrix(0, num_theta, num_replicates)

for (j in 1:num_theta){
  theta <- theta_seq[j]
  for (i in 1:num_replicates){
    y <- rbinom(num_indiv_samples, 1, theta)
    z_mat <- matrix(rbinom(num_pooled_samples * pool_size, 1, theta),
                    nrow = num_pooled_samples, ncol = pool_size)
    z <- as.numeric(rowSums(z_mat) > 0)

    y_samples <- find_theta_y(y)
    y_replicates[j,i,] <- y_samples$interval
    y_mean[j,i] <- y_samples$mean
    y_width[j,i] <- diff(y_samples$interval)

    z_samples <- find_theta_z(z_mat, 10000, .1)
    z_replicates[j,i,] <- z_samples$interval
```

```r
    z_mean[j,i] <- mean(z_samples$theta)
    z_width[j,i] <- diff(z_samples$interval)

    yz_samples <- find_theta_yz(y,z_mat, 10000, .1)
    yz_replicates[j,i,] <- yz_samples$interval
    yz_mean[j,i] <- mean(yz_samples$theta)
    yz_width[j,i] <- diff(yz_samples$interval)
  }
}

f1a_dat <- tibble(ci_width = c(y_mean, z_mean, yz_mean),
         prevalence = rep(theta_seq, 3 * num_replicates),
         Method = rep(c('individual samples','pooled only','data integration'),
                      each = num_theta * num_replicates))

f1a_mean <- f1a_dat %>%
  group_by(prevalence, Method) %>%
  summarise(width = mean(ci_width),.groups = 'drop')

f1a <- f1a_dat %>%
  ggplot(aes(y=ci_width, x = prevalence, color = Method)) +
  geom_point(alpha = .01) + theme_bw() +
  geom_line(aes(y = width, x = prevalence, color = Method), inherit.aes = F, data = f1a_mean) +
  ggtitle('Posterior Mean by Prevalence')  +
  ylab('Estimated prevalence') + xlab("prevalence (p)") +
  facet_wrap(.~Method) + theme(legend.position='none') +
  theme(axis.text.x = element_text(angle = 30))


f1b_dat <- tibble(ci_width = c(y_width, z_width, yz_width),
         prevalence = rep(theta_seq, 3 * num_replicates),
         Method = rep(c('individual samples','pooled only','data integration'),
                      each = num_theta * num_replicates))

f1b_mean <- f1b_dat %>%
  group_by(prevalence, Method) %>%
  summarise(width = mean(ci_width), .groups = 'drop')

f1b <- f1b_dat %>%
  ggplot(aes(y=ci_width, x = prevalence, color = Method)) +
  geom_point(alpha = .01) + theme_bw() +
  geom_line(aes(y = width, x = prevalence, color = Method), inherit.aes = F, data = f1b_mean) +
  ggtitle('Credible Interval Width by Prevalence') + ylab('Width') +
  xlab("prevalence (p)") + theme(legend.position='bottom')

grid.arrange(f1a,f1b)
```

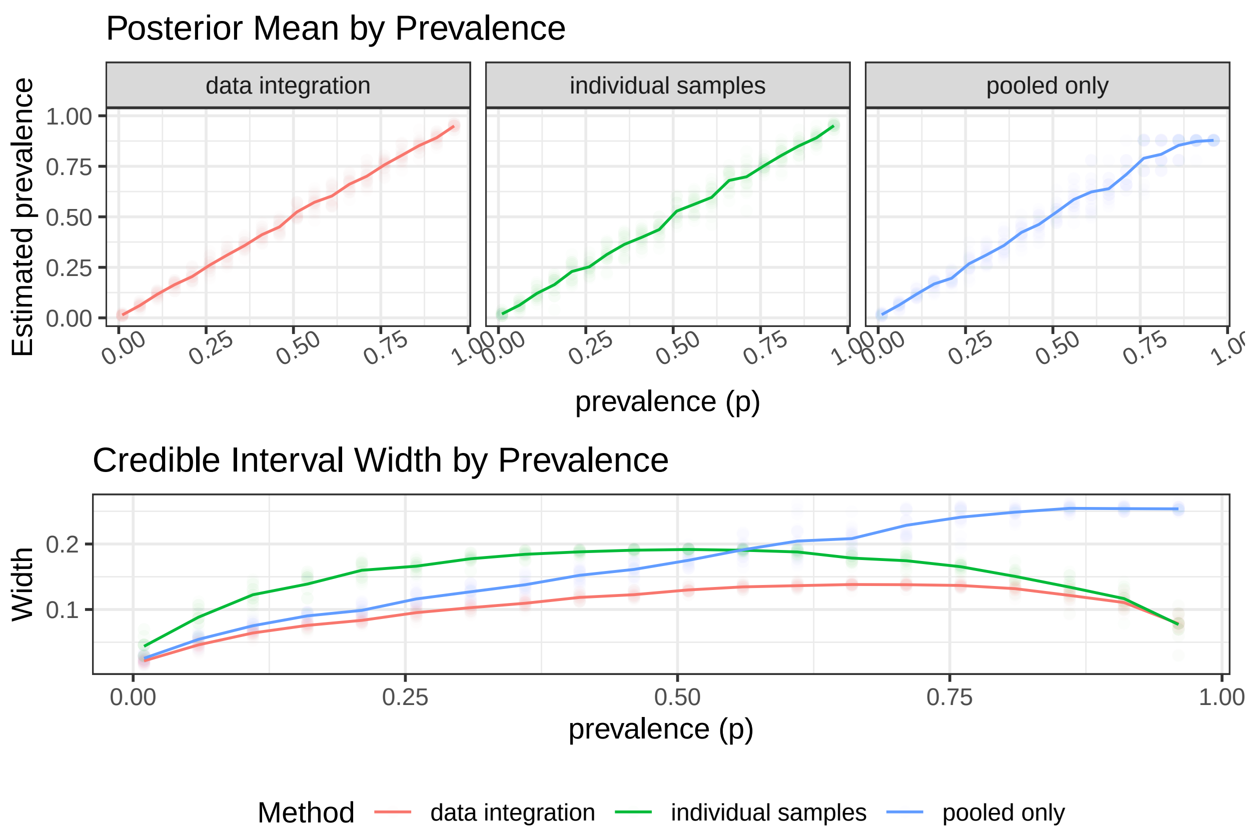

Part B: Varying N

```
num_pooled_samples <- c(10,50,100,500)
num_indiv_samples <- c(10,50,100,500)
k <- length(num_pooled_samples)
pool_size <- 3
num_theta <- length(theta_seq)

y_replicates <- array(0, dim = c(num_theta,num_replicates, 2,k))
z_replicates <- array(0, dim = c(num_theta,num_replicates, 2,k))
yz_replicates <- array(0, dim = c(num_theta,num_replicates, 2,k))
y_mean <- y_width <- array(0, dim =c(num_theta, num_replicates,k))
z_mean <- z_width <- array(0, dim = c(num_theta, num_replicates, k))
yz_mean <- yz_width <- array(0, dim = c(num_theta, num_replicates, k))

for (j in 1:num_theta){
  theta <- theta_seq[j]
  for (i in 1:num_replicates){
    for (n_val in 1:k){
      y <- rbinom(num_indiv_samples[n_val], 1, theta)
      z_mat <- matrix(rbinom(num_pooled_samples[n_val] * pool_size, 1, theta),
                      nrow = num_pooled_samples[n_val], ncol = pool_size)
      z <- as.numeric(rowSums(z_mat) > 0)

      y_samples <- find_theta_y(y)
      y_replicates[j,i,,n_val] <- y_samples$interval
```

```
      y_mean[j,i,n_val] <- y_samples$mean
      y_width[j,i,n_val] <- diff(y_samples$interval)

      z_samples <- find_theta_z(z_mat, 10000, .1)
      z_replicates[j,i,,n_val] <- z_samples$interval
      z_mean[j,i,n_val] <- mean(z_samples$theta)
      z_width[j,i,n_val] <- diff(z_samples$interval)

      yz_samples <- find_theta_yz(y,z_mat, 10000, .1)
      yz_replicates[j,i,,n_val] <- yz_samples$interval
      yz_mean[j,i,n_val] <- mean(yz_samples$theta)
      yz_width[j,i,n_val] <- diff(yz_samples$interval)
    }
  }
}


f1b_dat <- tibble(ci_width = c(y_width, z_width, yz_width),
         prevalence = rep(rep(theta_seq, 3 * num_replicates),k),
         n = paste('n = ', rep(rep(num_pooled_samples,each = num_replicates * length(theta_seq)),3)),
         Method = rep(rep(c('individual samples','pooled only','data integration'),
                      each = num_theta * num_replicates),each =k))

f1b_mean <- f1b_dat %>%
  group_by(prevalence, Method, n) %>%
  summarise(width = mean(ci_width), .groups = 'drop')

f1b_dat %>%
  ggplot(aes(y=ci_width, x = prevalence, color = Method)) +
  geom_point(alpha = .01) + theme_bw() +
  geom_line(aes(y = width, x = prevalence, color = Method), inherit.aes = F, data = f1b_mean) +
  ggtitle('Credible Interval Width by Prevalence') + ylab('Width') +
  xlab("prevalence (p)") + theme(legend.position='bottom') +
  facet_wrap(.~n)
```

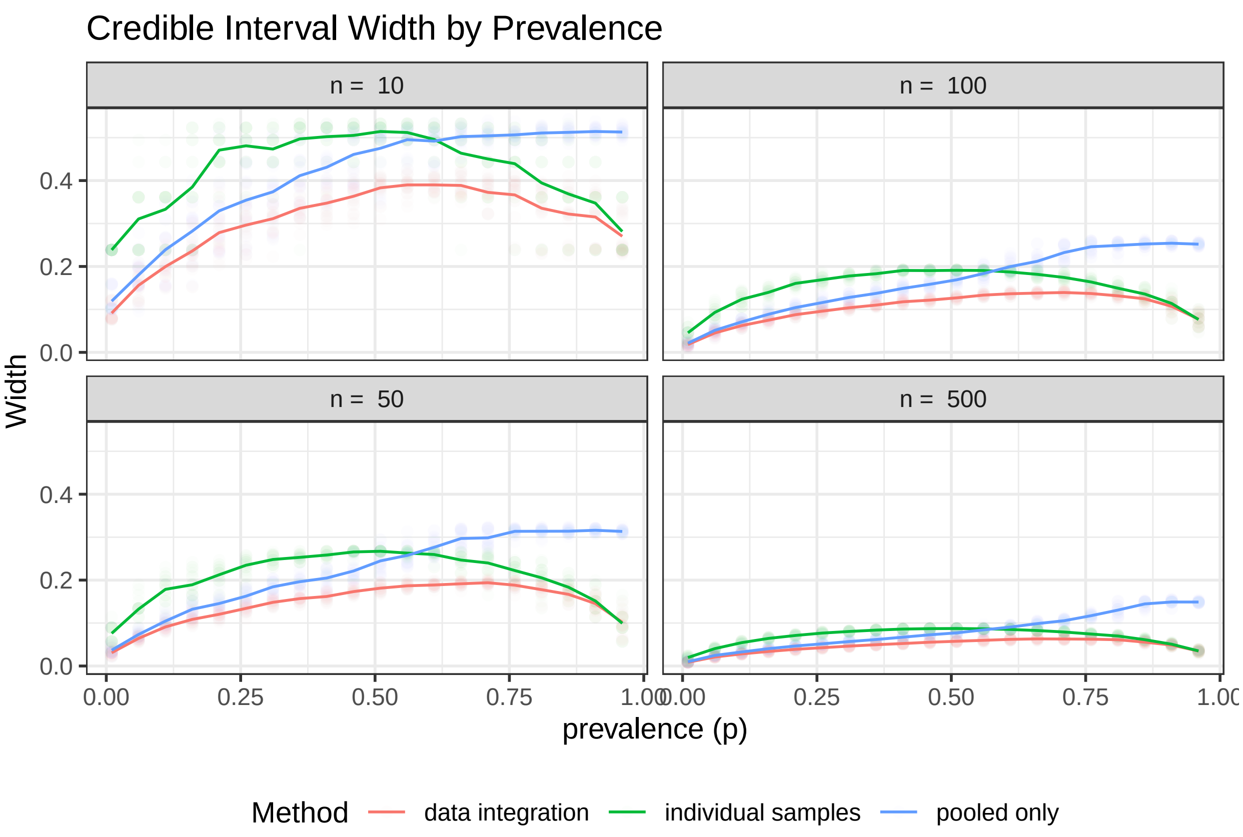

Simulation 2

Part A: fixed n = 100

```
set.seed(08012020)
num_pooled_samples <- 100
pool_size <- 5
num_theta <- length(theta_seq)

## Simulate and Fit Data
followup_mean <- followup_width <- integrate_mean <- integrate_width <-
  array(0, dim = c(num_theta, num_replicates))

for (j in 1:num_theta){
  theta <- theta_seq[j]
  for (i in 1:num_replicates){
    y <- rbinom(num_pooled_samples * pool_size, 1, theta)
    z_mat <- matrix(y, nrow = num_pooled_samples, ncol = pool_size)
    y_tmp <- find_theta_y(y)
    followup_mean[j,i] <- y_tmp$mean
    followup_width[j,i] <- diff(y_tmp$interval)
    followup_samples <- sum(z_mat) * pool_size
    y2 <- rbinom(followup_samples, 1, theta)
    yz_tmp <- find_theta_yz(y2, z_mat, num_mcmc = 10000, step_size = .1)
    integrate_mean[j,i] <- mean(yz_tmp$theta)
    integrate_width[j,i] <- diff(yz_tmp$interval)
```

```
  }
}

f1_dat <- tibble(ci_mean = c(integrate_mean,followup_mean),
       replicate = rep(rep(1:num_replicates, each = num_theta),2),
       type = rep(c('integrate','follow up'), each = num_theta * num_replicates),
       prevalence = rep(theta_seq, num_replicates * 2))

f1_mean <- f1_dat %>% group_by(type, prevalence) %>%
  summarise(ci_mean= mean(ci_mean), .groups = 'drop')

f1 <- f1_dat %>%
  ggplot(aes(y=ci_mean, x = prevalence, color = type)) +
  geom_point(alpha = .01) +
  geom_line(aes(y = ci_mean, x = prevalence, color = type), inherit.aes = F, data = f1_mean) +
  theme_bw() + ggtitle('Posterior Mean by Prevalence')  +
  ylab("Posterior mean for prevalence (p)") + xlab("prevalence (p)") +
  theme(legend.position='none') + facet_wrap(.~type)

f2_dat <- tibble(ci_width = c(integrate_width,followup_width),
       replicate = rep(rep(1:num_replicates, each = num_theta),2),
       type = rep(c('integrate','follow up'), each = num_theta * num_replicates),
       prevalence = rep(theta_seq, num_replicates * 2))

f2_mean <- f2_dat %>% group_by(type, prevalence) %>%
  summarise(ci_width = mean(ci_width), .groups = 'drop')

f2 <- f2_dat %>%
  ggplot(aes(y=ci_width, x = prevalence, color = type)) +
  geom_point(alpha = .01) +
  geom_line(aes(y = ci_width, x = prevalence, color = type), inherit.aes = F, data = f2_mean) +
  theme_bw() + ggtitle('Credible Interval Width by Prevalence')  +
  ylab("Credible Interval Width") + xlab("prevalence (p)") +
  theme(legend.position='bottom')

grid.arrange(f1,f2)
```

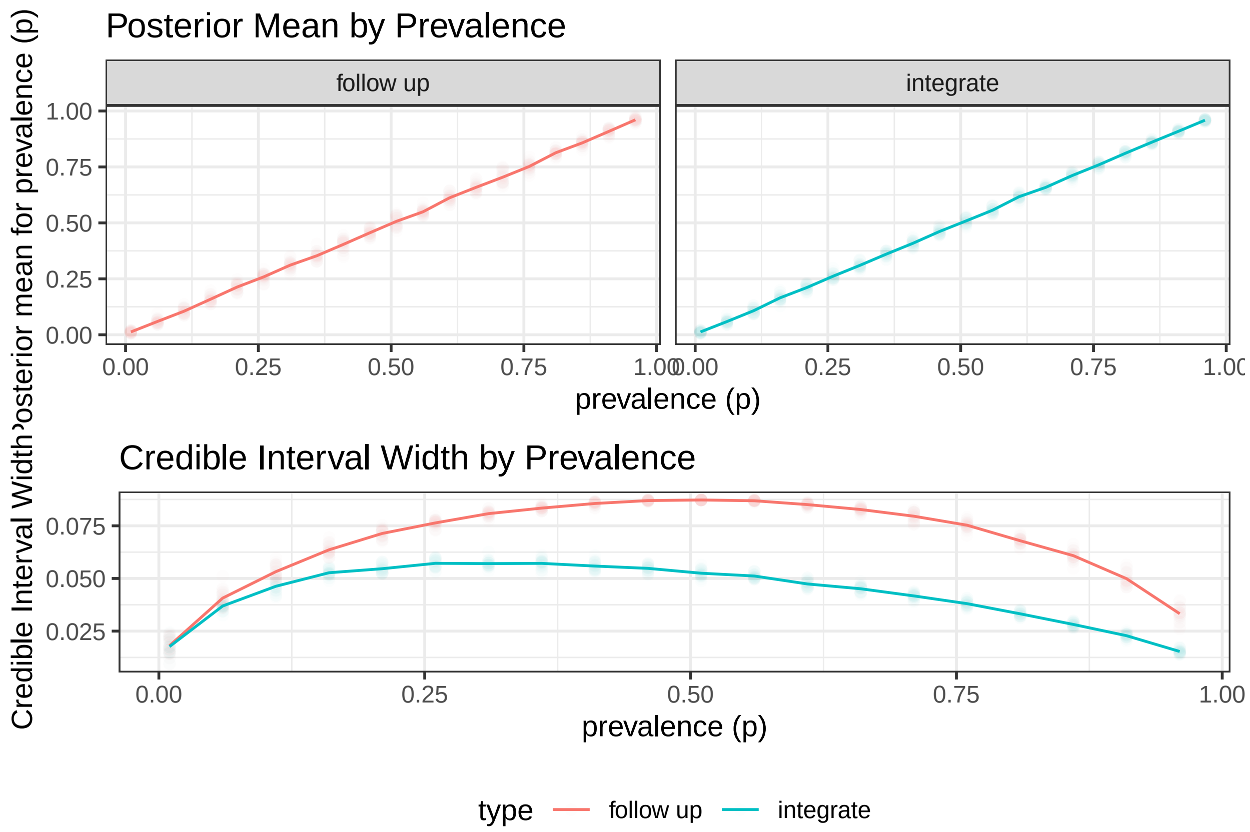

Part B: Varying N

```
set.seed(08012020)
num_pooled_samples <- c(10,50,100,500)
pool_size <- 5
num_theta <- length(theta_seq)
k <- length(num_pooled_samples)
## Simulate and Fit Data
followup_mean <- followup_width <- integrate_mean <- integrate_width <-
  array(0, dim = c(num_theta, num_replicates,k))

for (j in 1:num_theta){
  theta <- theta_seq[j]
  for (i in 1:num_replicates){
    for(k2 in 1:k){
      y <- rbinom(num_pooled_samples[k2] * pool_size, 1, theta)
      z_mat <- matrix(y, nrow = num_pooled_samples[k2], ncol = pool_size)
      y_tmp <- find_theta_y(y)
      followup_mean[j,i,k2] <- y_tmp$mean
      followup_width[j,i,k2] <- diff(y_tmp$interval)
      followup_samples <- sum(z_mat) * pool_size
      y2 <- rbinom(followup_samples, 1, theta)
      yz_tmp <- find_theta_yz(y2, z_mat, num_mcmc = 10000, step_size = .1)
      integrate_mean[j,i,k2] <- mean(yz_tmp$theta)
      integrate_width[j,i,k2] <- diff(yz_tmp$interval)
    }
```

```
  }
}

f2_dat <- tibble(ci_width = c(integrate_width,followup_width),
       replicate = rep(rep(rep(1:num_replicates, each = num_theta),2),k),
       type = rep(c('integrate','follow up'), each = num_theta * num_replicates * k),
       prevalence = rep(rep(theta_seq, num_replicates * 2),k),
       n = paste('n = ', rep(rep(num_pooled_samples, each = num_theta * num_replicates),2)))

f2_mean <- f2_dat %>% group_by(type, prevalence, n) %>%
  summarise(ci_width = mean(ci_width), .groups = 'drop')

f2_dat %>%
  ggplot(aes(y=ci_width, x = prevalence, color = type)) +
  geom_point(alpha = .01) +
  geom_line(aes(y = ci_width, x = prevalence, color = type), inherit.aes = F, data = f2_mean) +
  theme_bw() + ggtitle('Credible Interval Width by Prevalence')  +
  ylab("Credible Interval Width") + xlab("prevalence (p)") +
  theme(legend.position='bottom') + facet_wrap(.~n)
```

Credible Interval Width by Prevalence

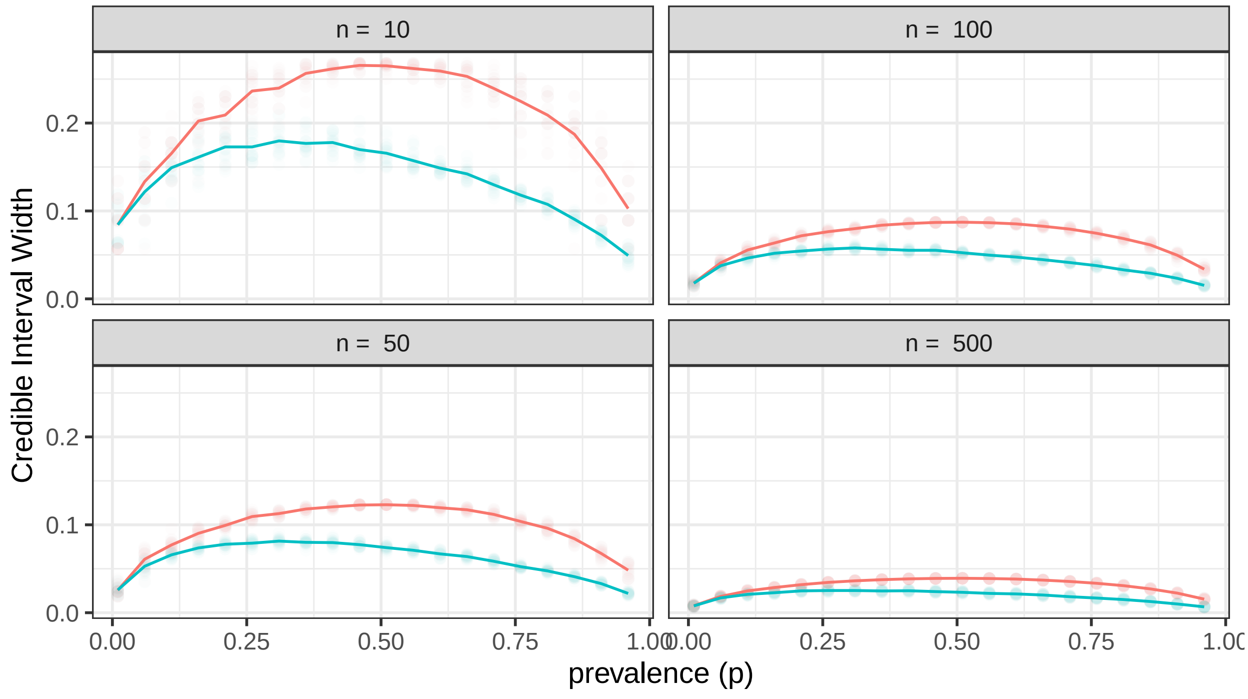

type — follow up — integrate

Simulation 3

```
set.seed(07052020)
num_pooled_samples <- 100
pool_size <- c(1,3,5,7,9)
pool_length <- length(pool_size)
```

```r
max_samples <- max(pool_size) * num_pooled_samples
num_theta <- length(theta_seq)

## Simulate and Fit Data
z_mean <- z_width <- array(0, dim = c(pool_length,num_theta, num_replicates))

for (j in 1:num_theta){
  theta <- theta_seq[j]
  for (i in 1:num_replicates){
    y <- rbinom(max_samples, 1, theta)
    for (k in 1:pool_length){
      z_mat <- matrix(y[1:(pool_size[k]*num_pooled_samples)],
                      nrow = num_pooled_samples, ncol = pool_size[k])
      z_samples <- find_theta_z(z_mat, 10000, .1)
      z_mean[k,j,i] <- mean(z_samples$theta)
      z_width[k,j,i] <- diff(z_samples$interval)
    }
  }
}


f1_dat <- tibble(ci_mean = c(z_mean),
       replicate = rep(1:num_replicates, each = pool_length * num_theta),
       num_pooled = factor(rep(pool_size, num_replicates * num_theta)),
       prevalence = rep(rep(theta_seq, each = pool_length),num_replicates))

f1_mean <- f1_dat %>% group_by(num_pooled, prevalence) %>%
  summarise(ci_mean = mean(ci_mean), .groups = 'drop')

f1 <- f1_dat %>% ggplot(aes(y=ci_mean, x = prevalence, color = num_pooled)) +
 geom_line(aes(y = ci_mean, x = prevalence, color = num_pooled), inherit.aes = F, data = f1_mean) +
  theme_bw() + ggtitle('Posterior Mean by Prevalence')  +
  ylab("Posterior mean for prevalence (p)") +
  xlab("prevalence (p)") + theme(legend.position='none')

f2_dat <- tibble(ci_mean = c(z_width),
       replicate = rep(1:num_replicates, each = pool_length * num_theta),
       `pool size` = factor(rep(pool_size, num_replicates * num_theta)),
       prevalence = rep(rep(theta_seq, each = pool_length),num_replicates))

f2_mean <- f2_dat %>% group_by(`pool size`, prevalence) %>%
  summarise(ci_mean = mean(ci_mean), .groups = 'drop')

f2 <- f2_dat %>% ggplot(aes(y=ci_mean, x = prevalence, color = `pool size`)) +
  geom_line(data = f2_mean) +
  theme_bw() + ggtitle('Credible Interval Width by Prevalence')  +
  ylab("Credible Interval Width") + xlab("prevalence (p)") +
  theme(legend.position='bottom')

grid.arrange(f1,f2)
```

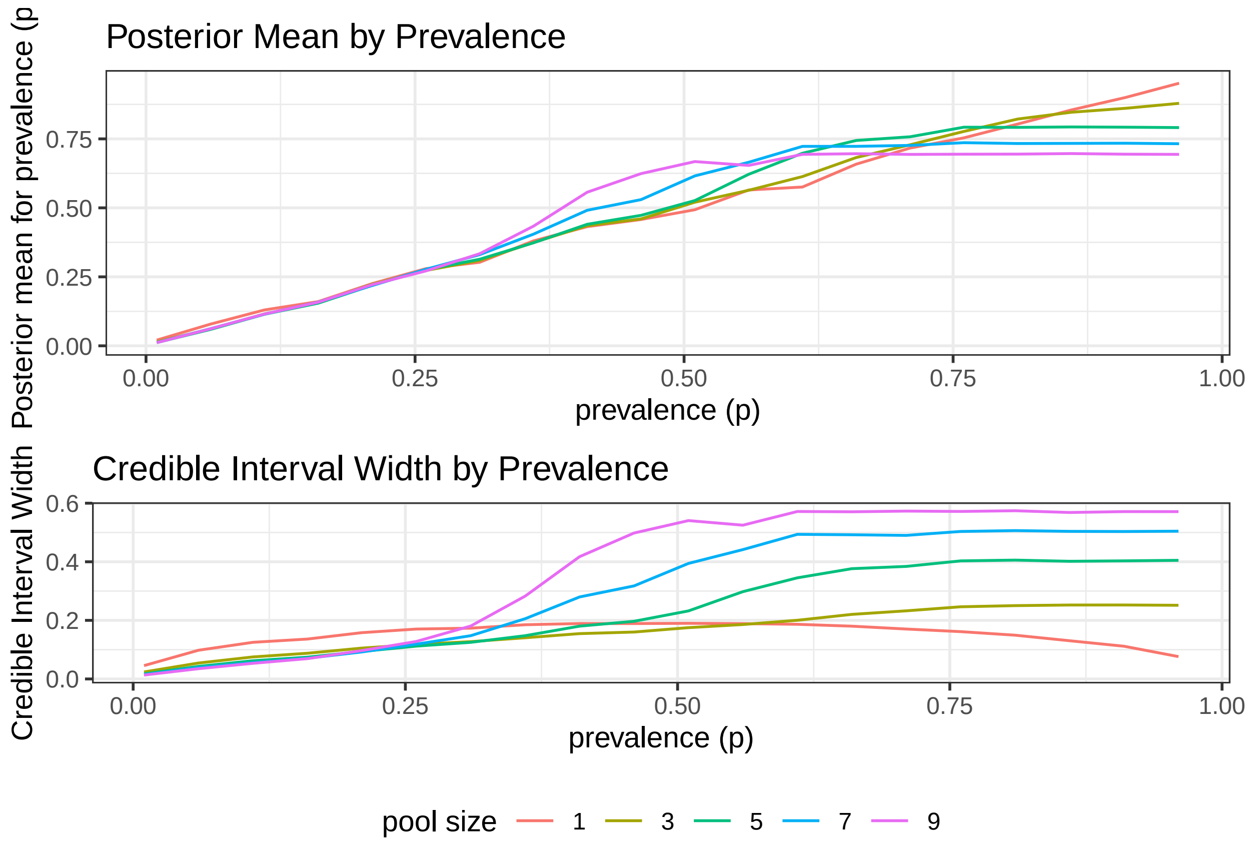

Simulation 4

```
####################################################
### Phase 1. Start with 20 samples of size 5.
####################################################
# initialize parameters
set.seed(07052020)
theta_length <- length(theta_seq)
num_theta <- length(theta_seq)
total_samples <- 100
pool_size <- 5
z <- array(0, dim = c(num_replicates, theta_length,
                      total_samples / pool_size, pool_size))
# Take Phase 1 Sample
z_mean <- z_width <- array(0, dim = c(num_replicates, theta_length))
for (i in 1:num_replicates){
  for (j in 1:theta_length){
    z[i,j,,] <- matrix(rbinom(total_samples,1,theta_seq[j]),
                       nrow = total_samples / pool_size, ncol = pool_size)
    z_samples <- find_theta_z(z[i,j,,], 10000, .1)
    z_mean[i,j] <- mean(z_samples$theta)
    z_width[i,j] <- diff(z_samples$interval)
  }
}
```

```r
################################################
### Phase 2. Take an additional 80 samples.
################################################

# a. Add individual samples
phase2_samples <- 80

y_width <- y_mean <- array(0, dim = c(num_replicates, theta_length))

for (i in 1:num_replicates){
  for (j in 1:theta_length){
    y <- rbinom(phase2_samples,1,theta_seq[j])
    y_samples <- find_theta_yz(y,z[i,j,,], 10000, .1)
    y_mean[i,j] <- mean(y_samples$theta)
    y_width[i,j] <- diff(y_samples$interval)
  }
}

tib1 <- tibble(vals = c(y_width),
               prevalence = rep(theta_seq, each = num_replicates))

# Add pooled samples of size 3
pool_size <- 3
z3_width <- z3_mean <- array(0, dim = c(num_replicates, theta_length))

for (i in 1:num_replicates){
  for (j in 1:theta_length){
    z3 <- matrix(rbinom(phase2_samples * pool_size,1,theta_seq[j]),
                 nrow = phase2_samples, ncol = pool_size)
    z_pooled <- as.numeric(c(rowMeans(z[i,j,,]) > 0, rowMeans(z3) > 0))
    n_pooled <- c(rep(5, nrow(z[i,j,,])), rep(pool_size, nrow(z3)))
    z3_samples <- find_theta_z2(z_pooled, n_pooled, 10000, .1)
    z3_mean[i,j] <- mean(z3_samples$theta)
    z3_width[i,j] <- diff(z3_samples$interval)
  }
}

tib3 <- tibble(vals = c(z3_width),
               prevalence = rep(theta_seq, each = num_replicates))


#Add pooled samples of size 5
pool_size <- 5
z5_width <- z5_mean <- array(0, dim = c(num_replicates, theta_length))

for (i in 1:num_replicates){
  for (j in 1:theta_length){
    z5 <- matrix(rbinom(phase2_samples * pool_size,1,theta_seq[j]),
                 nrow = phase2_samples, ncol = pool_size)
    z_pooled <- as.numeric(c(rowMeans(z[i,j,,]) > 0, rowMeans(z5) > 0))
    n_pooled <- c(rep(5, nrow(z[i,j,,])), rep(pool_size, nrow(z5)))
    z5_samples <- find_theta_z2(z_pooled, n_pooled, 10000, .1)
    z5_mean[i,j] <- mean(z5_samples$theta)
```

```
    z5_width[i,j] <- diff(z5_samples$interval)
  }
}

tib5 <- tibble(vals = c(z5_width),
              prevalence = rep(theta_seq, each = num_replicates))

f3a_dat <- tibble( vals = c(c(z_mean),c(y_mean), c(z3_mean),  c(z5_mean)),
                   theta = rep(rep(theta_seq, each = num_replicates),4),
                   method = rep(c('phase 1','phase 2 with 1', 'phase 2 with 3',
                                  'phase2 with 5'), each = num_replicates * theta_length))

f3a_mean <- f3a_dat %>% group_by(theta, method) %>%
  summarise(vals = mean(vals), .groups = 'drop')

f3a <- f3a_dat %>%
  ggplot(aes(y = vals, x = theta, color = method))  +
  geom_line(aes(y = vals, x = theta, color = method), inherit.aes = F, data = f3a_mean) +
  theme_bw() + ylim(0,1) + ggtitle('Posterior Mean by Strategy') +
  theme(legend.position = "none")  + ylab("Posterior mean for prevalence (p)") +
  xlab("prevalence (p)")

f3b_dat <- tibble( vals = c(c(z_width),c(y_width),  c(z3_width),  c(z5_width)),
                   theta = rep(rep(theta_seq, each = num_replicates),4),
                   method = rep(c('initial pool','phase 2 with 1', 'phase2 with 3',  'phase2 with 5'),
                             each = num_replicates * theta_length))

f3b_mean <- f3b_dat %>% group_by(theta, method) %>%
  summarise(vals = mean(vals), .groups = 'drop')

f3b <- f3b_dat %>%
  ggplot(aes(y = vals, x = theta, color = method)) + geom_line(data = f3b_mean) +
  theme_bw() + ylim(0,1) + ggtitle('Credible Interval Width by Strategy') +
  theme(legend.position='bottom') +  ylab("Credible Interval Width") +
  xlab("prevalence (p)")

grid.arrange(f3a, f3b)
```

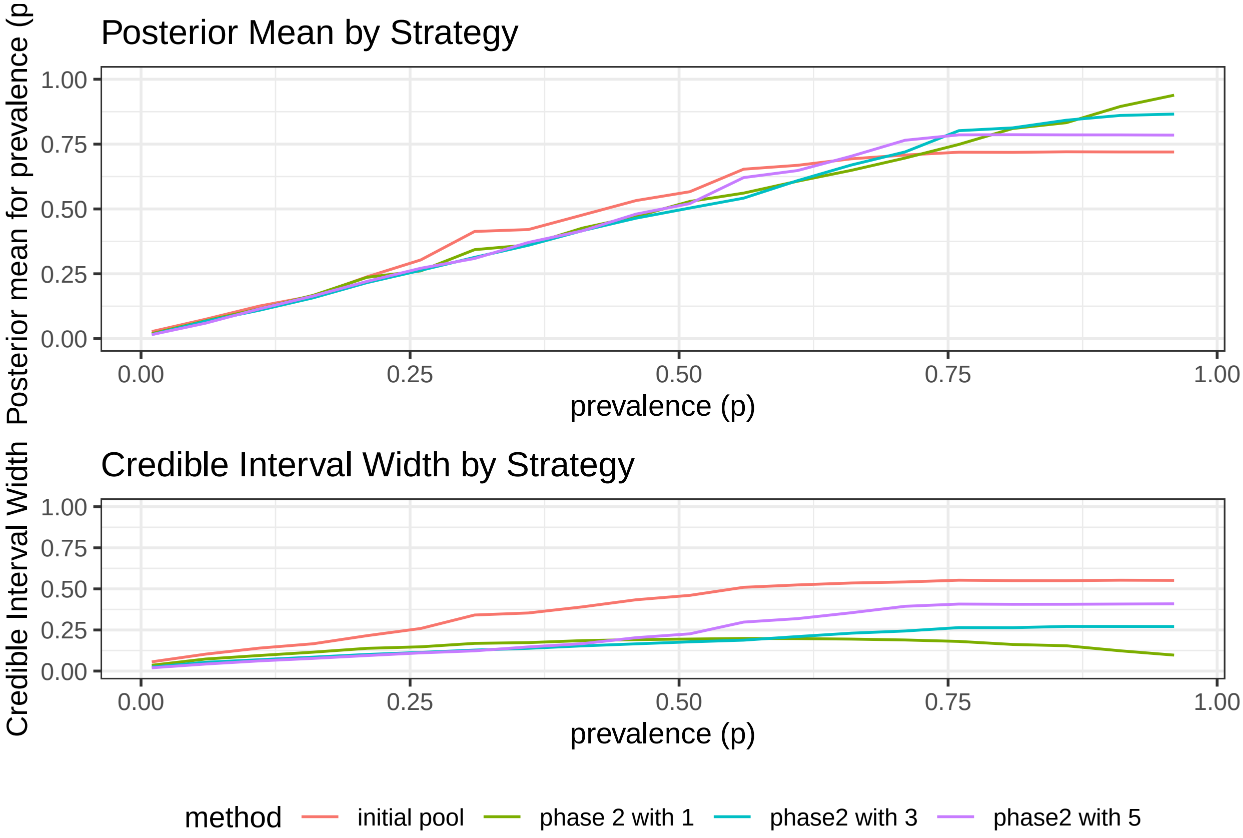
Posterior Mean by Strategy
Posterior mean for prevalence (p
1.00
0.75
0.50
0.25
0.00
0.00
0.25
0.50
0.75
1.00
prevalence (p)
Credible Interval Width by Strategy
Credible Interval Width
1.00
0.75
0.50
0.25
0.00
0.00
0.25
0.50
0.75
1.00
prevalence (p)
method
initial pool
phase 2 with 1
phase2 with 3
phase2 with 5
